# Name Him

**Published:** 1897-12

**Authors:** 


Name Him.—An editorial in the *Texas Medical News* for November—a very vigorous attack on a man up a tree, or a man in buckram—more than hints that there is a regular physician in Austin who is generally known to the community and to the medical profession, to make a practice of procuring abortions for a fee. It is universally agreed that the cat should be belled, but there it always stops. That such a thing should be true, known to be true, is a scandal on the profession, and the party ought to be exposed; but the how to do it is the rub, and nothing is done. Can the *Texas Medical News*, "the fox the finder," not uncover this villainy and bring the perpetrator to justice?

CERTAIN SPITEFUL REFERENCES to the editor of this journal as to his official connection with the quarantine department, which references need not be mentioned more specifically, will have to go unnoticed; their *animus* is perfectly well understood. He was indiscreet enough to kick a polecat once, several years ago, and he does not propose to subject myself to such another inundation of odoriferous rhetoric.

